# Validation of Doi’s weighted average glucose as a measure of post-load glucose excursion for clinical use

**DOI:** 10.17305/bb.2022.8807

**Published:** 2023-10-01

**Authors:** Saif Badran, Suhail A Doi, Atalla Hammouda, Omran A H Musa, Abdella M Habib

**Affiliations:** 1 Department of Population Medicine, College of Medicine, QU Health, Qatar University, Doha, Qatar; 2 Department of Plastic Surgery, Hamad General Hospital, Doha, Qatar; 3 Department of Basic Medical Sciences, College of Medicine, QU Health, Qatar University, Doha, Qatar

**Keywords:** Diabetes mellitus, oral glucose tolerance test (GTT), weighted average glucose, Doi’s weighted average glucose (dwAG), under the curve, homeostatic model assessment for insulin sensitivity (HOMA-S)

## Abstract

In this study, we examined the performance of a novel index of glucose excursion (Doi’s weighted average glucose [dwAG]) in relation to the conventional measure of area under the oral glucose tolerance test (A-GTT) and the homeostatic model assessment for insulin sensitivity (HOMA-S) and pancreatic beta cell function (HOMA-B). A cross-sectional comparison of the new index was conducted using 66 oral glucose tolerance tests (GTTs) performed at different follow-up times among 27 participants who had undergone surgical subcutaneous fat removal (SSFR). Comparisons across categories were made using box plots and the Kruskal–Wallis one-way ANOVA on ranks. Passing-Bablok regression was used to compare the dwAG against the conventional A-GTT. The Passing-Bablok regression model suggested a cutoff for normality for the A-GTT of 15.14 mmol/L⋅2h^−1^ compared to the dwAG’s suggested threshold of 6.8 mmol/L. For every 1 mmol/L⋅2h^−1^ increase in A-GTT, the dwAG value increased by 0.473 mmol/L. The glucose area under the curve correlated well with the four defined dwAG categories, with at least one of the categories having a different median A-GTT value (KW Chi2 ═ 52.8 [df ═ 3], *P* < 0.001). The HOMA-S tertiles were also associated with significantly different levels of glucose excursion measured through both the dwAG value (KW Chi2 ═ 11.4 [df ═ 2], *P* ═ 0.003) and A-GTT measure (KW Chi2 ═ 13.1 [df ═ 2], *P* ═ 0.001). It is concluded that the dwAG value and categories serve as a simple and accurate tool that can be used for interpreting glucose homeostasis across clinical settings.

## Introduction

Diabetes mellitus is a growing global pandemic that is increasing at an alarming rate. It is expected that diabetes prevalence will reach 10.2% (578 million), and that the prevalence of impaired glucose tolerance will reach 8% (454 million) by 2030 [1]. Half of the diabetic population has asymptomatic hyperglycemia [1] and this has led to further research on different diagnostic tools that can shed light on glycemic changes seen in patients with disorders of glucose homeostasis.

A test that has commonly been used to diagnose glycemic disorders is the oral glucose tolerance test (GTT), which is extensively used in both research and clinical practice as an indicator of gestational diabetes [2], but has been replaced by the fasting plasma glucose (FPG) for the diagnosis of type 2 diabetes [3]. In both humans and animals, the GTT provides an indication of the relative roles of insulin secretion and insulin resistance in the progression of glucose intolerance. It can provide the best measure of glucose homeostasis and has the potential to diagnose patients with impaired glucose tolerance even with normal FPG levels. This is of value because those patients are at higher risk for developing type 2 diabetes as well as cardiovascular diseases [4].

Doi’s weighted average glucose (dwAG) is a novel index that represents a single-value summary of the glucose excursion under the GTT. It is derived from only 3 time points on the GTT at 0, 60, and 120 min and was categorized into 4 levels in a previous study of gestational diabetes. These four categories differentiated between normal, impaired, abnormal, and severely abnormal glycemic states [2]. In this study, we examine the performance of the dwAG value in comparison to the area under the GTT (A-GTT) (75 mg oral glucose with 6 time points of glucose measurements) and homeostatic model assessment for insulin sensitivity (HOMA-S) and pancreatic beta cell function (HOMA-B) in a group of participants undergoing surgical subcutaneous fat removal for cosmetic purposes, also known as body contouring surgery. The aim was to determine whether the values and cutoffs as defined for gestational diabetes also define glucose excursion in a different group of adult subjects outside pregnancy.

## Materials and methods

### Subjects

We studied 27 consecutive eligible patients who underwent body contouring surgery at the Department of Plastic Surgery, Hamad General Hospital, in the period between July 2021 and June 2022. Sixteen participants were obese (59%) and 4 patients were diagnosed with type 2 diabetes mellitus (15%). Details of the participants are given in Table 1. GTT was performed at 3 different time points before and after surgery (visit one: within 1 week before surgery, visit two: 1 week after surgery, and visit three: 6 weeks after surgery). After taking a detailed medical history and complete physical examination, patients with comorbidities were excluded except for type 2 diabetes mellitus patients who were not on insulin therapy.

**Table 1:** Baseline characteristics of study participants

**Factor**	**Level/units**	**Visit 1**	**Visit 2**	**Visit 3**
		*N* ═ 27	*N* ═ 22	*N* ═ 19
Sex	Male	6 (22.22%)	6 (27.27%)	3 (15.79%)
	Female	21 (77.78%)	16 (72.73%)	16 (84.21%)
BMI category	Normal	4 (14.81%)	3 (13.64%)	2 (10.53%)
	Overweight	7 (25.93%)	6 (27.27%)	5 (26.32%)
	Obese	16 (59.26%)	13 (59.09%)	12 (63.16%)
Bariatric surgery status	No prior history	17 (62.96%)	13 (59.09%)	13 (68.42%)
	Had a prior history	10 (37.04%)	9 (40.91%)	6 (31.58%)
Fat percent, median (IQR)	%	37.00 (32.90, 42.20)	37.00 (32.90, 42.90)	39.60 (33.60, 44.00)
Biphasic shape of GTT	No	23 (85.19%)	19 (86.36%)	15 (88.24%)
	Yes	4 (14.81%)	3 (13.64%)	2 (11.76%)
Peak glucose after 30 min on the GTT	No	7 (25.93%)	5 (22.73%)	4 (23.53%)
	Yes	20 (74.07%)	17 (77.27%)	13 (76.475)
GTT0, median (IQR)	mmol/L	5.30 (4.90, 5.80)	5.45 (5.00, 5.70)	5.20 (4.90, 9.00)
GTT15, median (IQR)	mmol/L	7.90 (6.80, 9.70)	7.45 (7.00, 8.30)	8.00 (6.20, 9.00)
GTT30, median (IQR)	mmol/L	8.60 (7.10, 11.20)	8.40 (7.70, 10.30)	8.95 (6.80, 10.70)
GTT45, median (IQR)	mmol/L	10.40 (7.50, 12.00)	9.55 (8.40, 10.10)	8.10 (6.40, 10.70)
GTT60, median (IQR)	mmol/L	8.90 (7.20, 12.60)	8.75 (7.60, 11.10)	8.35 (7.10, 11.50)
GTT120, median (IQR)	mmol/L	6.70 (4.20, 8.80)	6.40 (5.30, 8.60)	5.75 (4.70, 8.20)

GTT: Glucose tolerance test; IQR: Interquartile range; BMI: Body mass index.

### Study design

The research design in this study was a cross-sectional comparison of standard and new method of assessing glucose excursion under the GTT. The GTT was administered using 75 mg oral glucose with 6 time points of glucose measurements (fasting [gtt0], 15 min [gtt15], 30 min [gtt30], 45 min [gtt45], 60 min [gtt60], and 120 min [gtt120] in mmol/L). For each of the GTT’s, glucose excursion was computed using:

a) standard method: Tai’s trapezoidal rule for the area under the GTT (A-GTT) [5] expressed as mmol/L/2h^−1^ using 6 GTT values (at 0, 15, 30, 45, 60, and 120 min)

b) new method: Doi’s weighted average glucose (dwAG) [2] calculated using the formula (gtt0×0.28)+(gtt60×0.36)+(gtt120×0.36) and expressed as actual glucose values in mmol/L. The dwAG represents a single-value summary of the glucose excursion under the GTT using only the 3 time points (0, 60, and 120 min) in routine GTT’s for diagnostic use [2]. The dwAG value was categorized into 4 categories: dwAG0 ≤ 6.8, dwAG1 > 6.8 and ≤ 7.5, dwAG2 > 7.5 and ≤ 8.6, and dwAG3 > 8.6 mmol/L based on 4 levels of risk previously defined for women with gestational diabetes [2]. These four levels of dwAG reflect normal, impaired, abnormal, and severely abnormal dwAG values, respectively.

The Oxford HOMA2 Calculator was used to compute HOMA-S and HOMA-B (both anchored at 100% for normal insulin sensitivity) using FPG and fasting C-peptide [6].

The GTTs were classified into two patterns or shapes that indicate a higher level of beta cell dysfunction:

a) Those that peaked after 30 min (Y/N) defined as a maximum value after 30 min (or after 45 min if the value at this time only exceeded the 30 min value by < 0.25 mmol/L) [7].
b) A biphasic GTT defined as a GTT with 120 min glucose ≥ 0.25 mmol/L higher than at 60 min [8].

### Ethical statement

All subjects signed an informed consent before starting the study, which was approved by the Institutional Review Board at Hamad Medical Corporation and Qatar University (MRC-01-20-466 and QU-IRB 1412-EA/20, respectively), and by the Institutional Bio-safety Committee at Qatar University (QU-IBC-2020/066).

### Statistical analysis

Comparisons across categories were made using box plots and the Kruskal–Wallis one-way ANOVA on ranks which extends the Mann–Whitney U test. Passing–Bablok regression was used to compare both methods of computing glucose excursion and is a linear regression procedure with no special assumptions regarding the distribution of the samples and the measurement errors [9]. The result does not depend on the assignment of the methods for glucose excursion to X and Y. A linear regression model with two categorical predictors (peak after 30 min and biphasic GTT) was used to assess mean values of dwAG, A-GTT, HOMA-S, and HOMA-B in groups defined by these factors. Finally, the dependence of dwAG on HOMA-S and HOMA-B was modeled in linear regression using restricted cubic splines and using the values of both HOMA-S and HOMA-B indices centered at 100%. Stata version 15 (College Station, TX, USA) was used for all analyses and exact *P* values were reported throughout.

**Figure 1.**
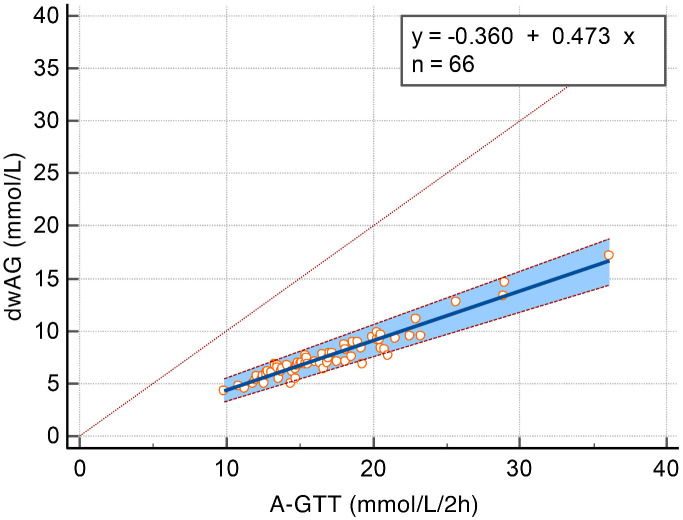
**Passing–Bablok regression plot showing data and fit for dwAG measure of glucose excursion predicted from A-GTT.** Cusum test for linearity, no significant deviation from linearity (*P* ═ 0.83); Spearman rank correlation coefficient 0.934 (95% CI 0.894–0.959). A-GTT: Area under the glucose tolerance test; dwAG: Doi’s weighted average glucose.

## Results

**Figure 2.**
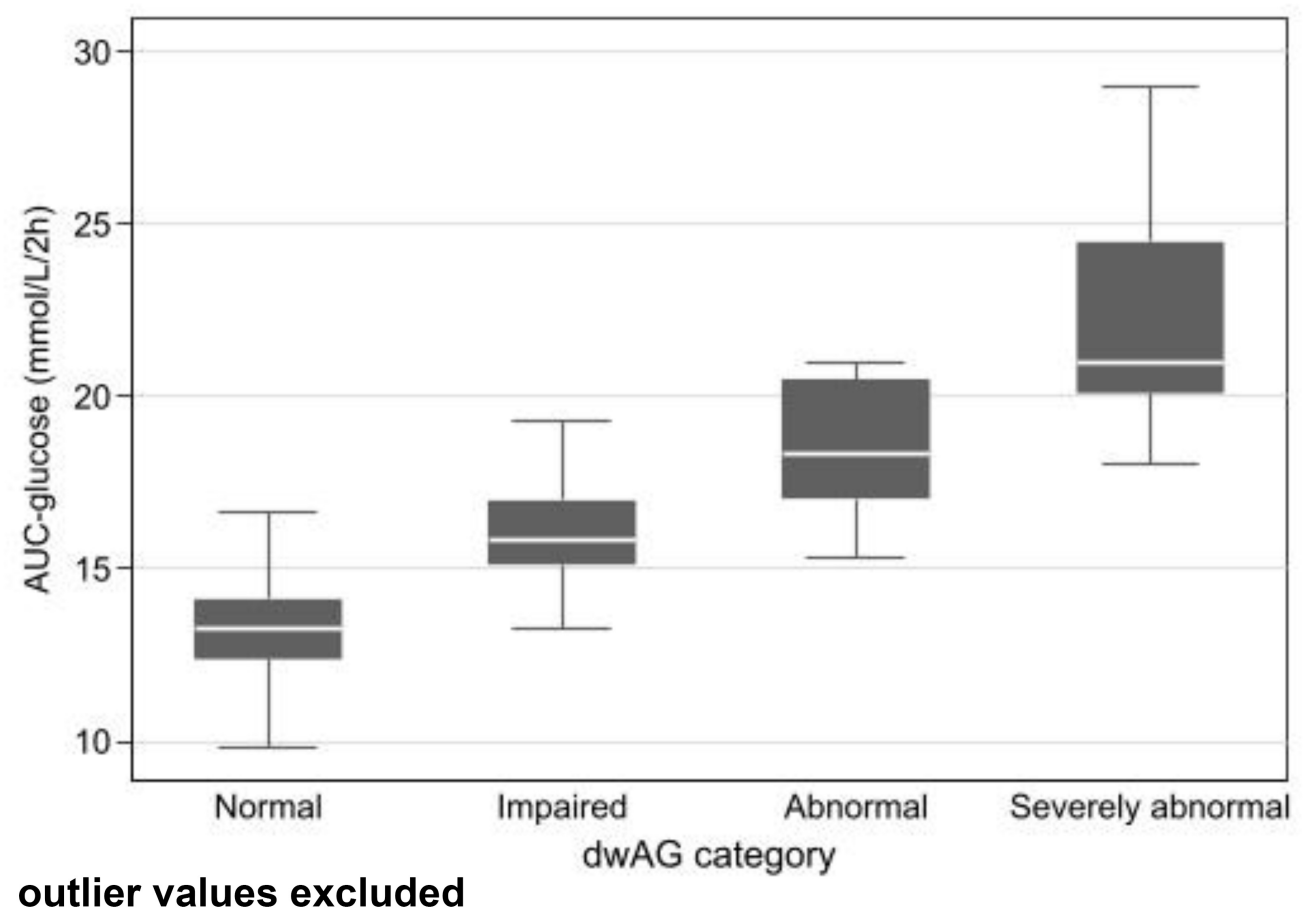
**Association between the A-GTT and dwAG category measure of glucose excursion.** A-GTT: Area under the glucose tolerance test; dwAG: Doi’s weighted average glucose.

There was a total of 66 complete GTTs and of these, 47 (71.2%) had peak values after 30 min and 9 (13.6%) were biphasic (8/9 also had a peak after 30 min). Glucose excursion was computed using the two measures indicated in the methods and a Passing–Bablok regression model suggested a cutoff for normal values for the A-GTT of 15.14 mmol/L⋅2h^−1^ as the equivalent cutoff to the dwAG value of 6.8 mmol/L. For every 1 mmol/L⋅2h^−1^ increase in A-GTT, the dwAG value increased by 0.473 mmol/L (Figure 1). The glucose area under the curve correlated well with the dwAG level (4 groups), with at least one of the levels having a different A-GTT (KW Chi2 ═ 52.8 [df ═ 3], *P* < 0.001). The median A-GTT in each dwAG category was 13.2, 15.9, 18.3, and 21.0 mmol/L⋅2h^-1^ in the normal, impaired, abnormal, and severely abnormal dwAG groups, respectively (Figure 2).

**Figure 3.**
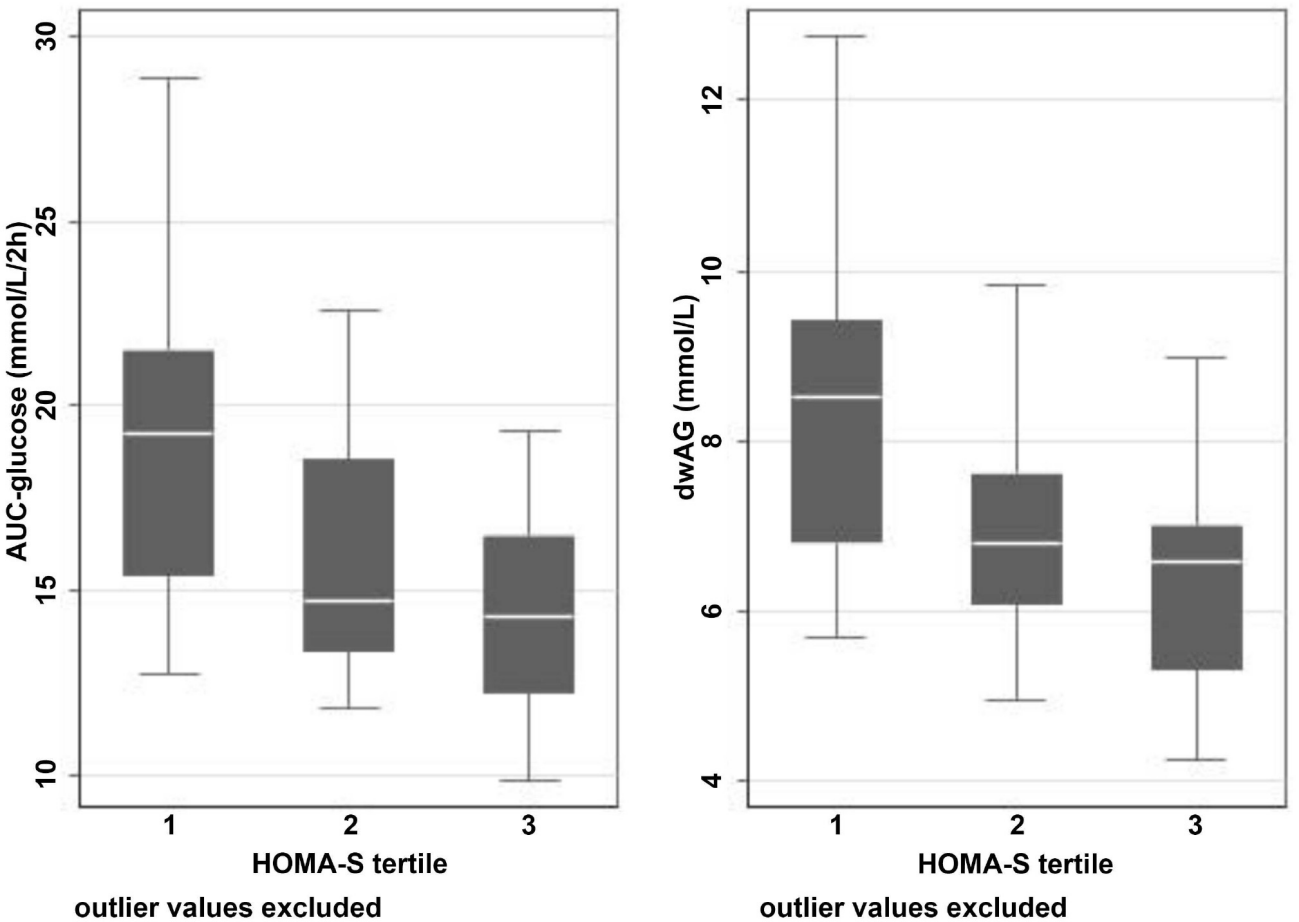
**Association between glucose excursion (left: Tai’s A-GTT; right: dwAG) and HOMA-S tertile.** HOMA-S tertile: Homeostatic Model Assessment for insulin sensitivity tertiles 1, 2, and 3; A-GTT: Area under the glucose tolerance test; dwAG: Doi’s weighted average glucose.

The HOMA-S tertiles were associated with different levels of glucose excursion measured through both the dwAG value (KW Chi2 ═ 11.4 [df ═ 2], *P* ═ 0.003) and A-GTT value (KW Chi2 ═ 13.1 [df ═ 2], *P* ═ 0.001) (Figure 3). The IQRs for the dwAG across the insulin sensitivity tertiles were 6.8–9.4, 6.1–7.6, and 5.3–7.0 mmol/L, respectively. For A-GTT, the IQRs were 15.4–21.5, 13.3–18.5, and 12.2–16.5 mmol/L⋅2h^−1^, respectively. The impact on glucose excursion was seen more prominently once insulin sensitivity was lowest (in the first tertile; HOMA-S median −53.35%, IQR −69.7% to −49.1%).

**Figure 4.**
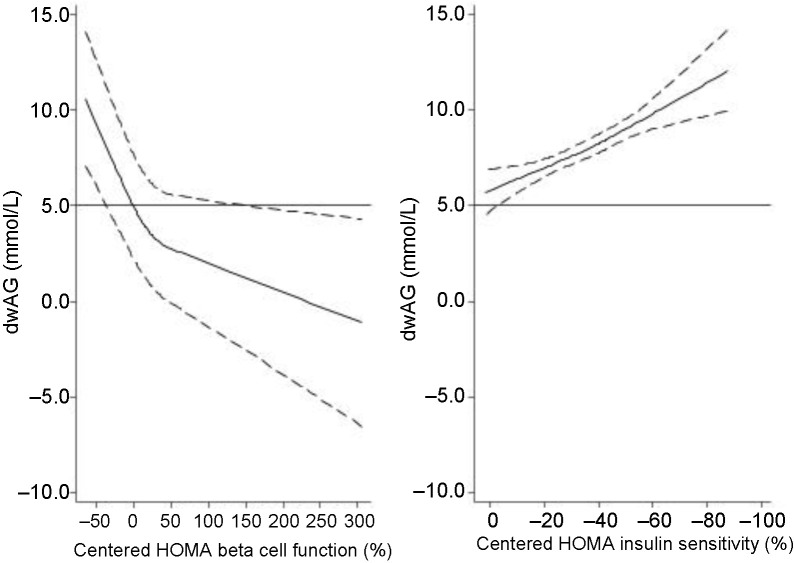
**Relationship of HOMA insulin sensitivity (%) and HOMA beta cell function (%) with the dwAG (mmol/L) demonstrating that both are important in determining the dwAG value.** The values of HOMA are centered at 100%, and when both are normal (0%), the dwAG value is about 5 mmol/L. As HOMA insulin sensitivity decreases, the dwAG rises linearly and this is mitigated by an increase in beta cell function which drops the dwAG value. dwAG: Doi’s weighted average glucose; HOMA: Homeostatic model assessment.

HOMA-B alone (in a non-linear regression model) explained 42% of the variation in dwAG values, whereas HOMA-S explained 9% of the variation in dwAG values in a similar model. The combination of both HOMA-B and HOMA-S in a non-linear regression model (using restricted cubic splines) contributed to explaining 66% of the variation in dwAG values suggesting that the combination was what defined the bulk of the variation in dwAG values. This is shown graphically in Figure 4, which shows that dwAG depends on both beta cell function and insulin sensitivity, and that dwAG increases as both insulin sensitivity and beta cell function decline.

The mean dwAG value in those GTTs with a peak at 30 min and that were monophasic was 6.4 mmol/L. There was a mean increase in dwAG of 1.4 mmol/L (*P* ═ 0.032) in those GTTs with a peak after 30 min but no biphasic shape and a mean increase of 3.0 mmol/L in GTTs with both a later peak and biphasic shape (*P* ═ 0.002). With the A-GTT the mean changes followed a similar trend but with less statistical evidence against the model hypothesis at this sample size. HOMA-B declined on average by 19.8% (*P* ═ 0.262) in the late peak-only group and by 29.3% (*P* ═ 0.285) in the combined late peak and biphasic group. The respective changes in HOMA-S for these groups were −3% and −3.7%, respectively, suggesting that these shape changes reflected beta cell function. None of the GTTs from a patient with a history of bariatric surgery demonstrated a biphasic pattern (*P* ═ 0.028, Fisher’s exact test), but a peak after 30 min occurred with equal frequency in those with or without a history of bariatric surgery.

## Discussion

This study, for the first time, introduces a novel tool to define glucose homeostasis in adult population, demonstrates an excellent correlation with the A-GTT, and is well discriminated by tertiles of HOMA-S. The implication here is that the dwAG, which combines fasting, 1h and 2h plasma glucose values, is a sufficient criterion for measuring glucose excursion in adults, which in this paper refers specifically to a group of non-pregnant adults who underwent body contouring surgery. The dwAG was responsive to GTTs with peaks after 30 min or with a biphasic shape and this was not so clearly evident with the A-GTT. The time to glucose peak >30 min has previously been shown to be an independent indicator of prediabetes and lower beta cell function in an otherwise healthy multi-ethnic adult cohort [7]. It is known that the glucose peak occurs most frequently at 30 min (60.5%) and is accompanied by a synchronous peak of insulin [10]. Thus, both a later peak and a biphasic shape indicate worse beta cell function [8, 11].

It was noted that none of the GTTs showed a biphasic pattern in subjects who had a history of bariatric surgery, which is not surprising given the fact that meal-induced secretion of glucagon-like peptide-1 (GLP-1) could be up to 10-fold higher in patients after gastric bypass or sleeve gastrectomy surgeries compared to non-surgical individuals and leads to an improvement in beta cell function in the longer term. One possible mechanism for this increase (among others) that has been put forward is accelerated nutrient transit from the stomach to the gut, leading to enhanced secretion of GLP-1 [12]. Increased food transit is specific to bariatric surgery and occurs before weight loss; given that our patients had bariatric surgery more than 18 months preceding the GTT, the favorable effect on beta cell function is sustained. This is different from the favorable effects of bariatric surgery on peripheral insulin sensitivity which is shared with those of calorie restriction [13] and is only improved in proportion to weight loss [14, 15].

The implication from the observations in these patients with or without history of bariatric surgery is that the measure of glucose excursion using the dwAG value shares elements of the two major metabolic impairments associated with glucose homeostasis: an increase in insulin resistance and impaired beta cell function [16, 17]. This was demonstrated in this study (Figure 4), where the dwAG was about 5 mmol/L when both HOMA indices were normal (100%). There was a linear increase in dwAG when insulin sensitivity declined and a non-linear increase in dwAG when beta cell function declined. This explains why the dwAG or A-GTT curve may be a better indicator of transitioning to type 2 diabetes mellitus [18] or future cardiovascular disease and mortality [19] than low insulin sensitivity alone. As indicated in our results, the dwAG correlates better with shape parameters than A-GTT and this was evident in post-bariatric subjects with much better beta cell function.

This study provides firm support to the dwAG as an alternative and novel method to formally assess glucose excursion under the GTT. Although it was developed for gestational diabetes [2], it is shown here that it can have broader application. This study confirms that the same groupings from normal to severely abnormal glucose excursion hold in this population of adults outside of pregnancy and, as expected, correlates with HOMA insulin sensitivity and beta cell function. While the GTT is no longer used as a mainstay in diabetes diagnosis [20, 21], it continues to be used as an index of glucose excursion, which represents the balance of insulin sensitivity and beta cell function. While those with abnormal dwAG values (dwAG2) had a mean FPG of 5.6 mmol/L which coincides with the ADA threshold for impaired fasting glucose (IFG), these two tests, nevertheless, reflect different levels of glucose homeostasis assessment because the GTT combines information from both insulin sensitivity and beta cell function [22], whereas the FPG is responsive primarily to insulin secretion relative to the level of insulin resistance [23] and is ideally suited to diabetes diagnosis as it indicates decompensated insulin resistance.

The strengths of the present study include a first-time comparison of the A-GTT to a novel index of glucose excursion using the conventional GTT used in clinical practice, the computation of the A-GTT from six time points of the GTT, and the comparison of both the conventional and novel indices to HOMA beta cell function and insulin sensitivity in the same model. Potential limitations include the fact that we have not yet acquired data on various hormones of interest during GTT (which is currently ongoing) and the use of a single GTT for the comparisons in individuals, which may have less reproducibility but, on the positive side, mimics the clinical use of these indices in practice.

## Conclusion

The dwAG represents a single-value summary of glucose excursion under the GTT and serves as a simple but accurate tool that can be used for glucose homeostasis interpretation. It was initially concieved as a tool that could be used to define glucose homeostasis in pregnancy and, in that study, correlated with adverse perinatal outcomes. It has now been independently validated as equivalent to the conventional A-GTT (based on six time points) measure of glucose excursion in this study in a different population of non-pregnant adults and correlates well with both HOMA-S and HOMA-B.
